# Structural Insights Into the Transcriptional Regulation of HigBA Toxin–Antitoxin System by Antitoxin HigA in *Pseudomonas aeruginosa*

**DOI:** 10.3389/fmicb.2019.03158

**Published:** 2020-01-22

**Authors:** Ying Liu, Zengqiang Gao, Guangfeng Liu, Zhi Geng, Yuhui Dong, Heng Zhang

**Affiliations:** ^1^Beijing Synchrotron Radiation Facility, Institute of High Energy Physics, Chinese Academy of Sciences, Beijing, China; ^2^National Center for Protein Science Shanghai, Shanghai Advanced Research Institute, Chinese Academy of Sciences, Shanghai, China; ^3^University of Chinese Academy of Sciences, Beijing, China

**Keywords:** toxin–antitoxin system, HigB-HigA, transcription regulator, crystal structure, DNA-binding protein

## Abstract

HigB-HigA is a bacterial toxin–antitoxin (TA) system in which the antitoxin HigA can mask the endoribonuclease activity of toxin HigB and repress the transcription of the TA operon by binding to its own promoter region. The opportunistic pathogen *Pseudomonas aeruginosa* HigBA (PaHigBA) is closely associated with the pathogenicity by reducing the production of multiple virulence factors and biofilm formation. However, the molecular mechanism underlying HigBA TA operon transcription by PaHigA remains elusive. Here, we report the crystal structure of PaHigA binding to the promoter region of *higBA* operon containing two identical palindromic sequences at 3.14 Å resolution. The promoter DNA is bound by two cooperative dimers to essentially encircle the intact palindrome region. The helix-turn-helix (HTH) motifs from the two dimers insert into the major grooves of the DNA at the opposite sides. The DNA adopts a canonical B-DNA conformation and all the hydrogen bonds between protein and DNA are mediated by the DNA phosphate backbone. A higher resolution structure of PaHigA-DNA complex at 2.50 Å further revealed three water molecules bridged the DNA-binding interface and mediated the interactions between the bases of palindromic sequences and PaHigA (Thr40, Asp43, and Arg49). Structure-based mutagenesis confirmed these residues are essential for the specific DNA-binding ability of PaHigA. Our structure–function studies therefore elucidated the cooperative dimer–dimer transcription repression mechanism, and may help to understand the regulation of multiple virulence factors by PaHigA in *P. aeruginosa*.

## Introduction

Toxin–antitoxin (TA) loci are small genetic modules that are widespread in bacterial plasmids and chromosomes and target various cellular functions to regulate cell growth and death (Gerdes et al., 2005; Yamaguchi and Inouye, 2011). TA systems have been characterized into six different types (types I–VI) based on the interaction mode of the TA and the molecular nature of the antitoxin (Page and Peti, 2016). Type II TA is the most well-characterized and abundant system, in which an antitoxin can bind directly to a cognate toxin to form a tight protein-protein complex for inactivating toxicity during normal growth. The type II antitoxins are generally composed of a DNA-binding domain (usually intrinsically disordered) neighboring a toxin-neutralizing domain (Loris and Garcia-Pino, 2014). The DNA-binding domain can autoregulate TA operon transcription by binding to its own promoter region.

HigB-HigA is a bacterial Type II TA system found in many pathogens, such as *Pseudomonas aeruginosa, Proteus vulgaris, Vibrio cholerae*, and *Mycobacterium tuberculosis* (Kędzierska and Hayes, 2016). The antitoxin gene *higA* and the toxin gene *higB* are located in one operon and share one promoter that is upstream of *higB*-*higA* pair. HigA can bind to the promoter region to autoregulate the transcription of this toxin-antitoxin operon. This system has an unusual gene arrangement, in which the toxin gene *higB* is upstream of the antitoxin gene *higA*. Such arrangement has also been observed in some other TA modules such as *mqsRA*, *hicAB*, and *brnTA* (Jorgensen et al., 2009; Yamaguchi et al., 2009; Heaton et al., 2012). The toxin HigB belongs to RelE toxin superfamily, which also include MqsR, BrnT, YafQ and YoeB subfamilies. These toxins are ribosome-dependent and cleave mRNAs preferentially at stop codons in the ribosomal A site (Pedersen et al., 2003; Hurley and Woychik, 2009; Schureck et al., 2015, 2016). The antitoxin HigA can mask the endoribonuclease activity of toxin HigB and repress the transcription of the TA operon by binding to its own promoter region. All the known HigB-HigA complex structures display a hetero-tetramer with the organization of HigB-(HigA)_2_-HigB, where HigA does not mask the putative catalytic cleft of HigB (Schureck et al., 2014; Yang et al., 2016; Hadži et al., 2017; Yoon et al., 2019). HigA consist of five α helices with a canonical helix-turn-helix (HTH) DNA-binding fold, and HigB displays a RelE-type ribonuclease fold consistent with the RelE/YoeB family.

HigBA is prevalent in the opportunistic pathogen *P. aeruginosa* clinical isolates (Williams et al., 2011; Andersen et al., 2017), and several recent studies have elucidated its physiological roles related to the pathogenicity. Wood et al. first found that activation of toxin HigB may influence several virulence factors, including pyocyanin and pyochelin as well as swarming motility and biofilm formation (Wood and Wood, 2016). Zhang et al. (2018) further found that HigB can decrease the intracellular level of c-di-GMP, which is responsible for the increased expression of type III secretion system (T3SS) genes and repression of biofilm formation (Li et al., 2016). A recent study showed PaHigA can function as a transcription repressor to control the virulence gene *mvfR* (multiple virulence factor regulator) expression besides its autoregulation of HigBA TA operon (Guo et al., 2019). The promoter analysis identified one and two identical palindromic sequences (5′-TTAAC GTTAA-3′) in the promoter region of *mvfR* gene and *higBA* operon, respectively. PaHigA can specifically bind to the promoter of the *mvfR* gene containing the palindromic sequence to reduce the synthesis of the pyocyanin virulence factor.

Despite the importance of HigBA TA system regulation in persistence and biofilms in *P. aeruginosa*, very little is known about how HigA binds and recognizes the promoter DNA at a molecular level. In this study, we reported the structures of PaHigA bound to the promoter region of the *higBA* operon. The structure showed that this antitoxin represses gene expression by a probably cooperative binding of two dimers. The HTH motifs from the four protomers insert into the major grooves of the DNA symmetrically. Structure-based mutagenesis on the interacting residues in the HTH motifs confirmed their essential roles in DNA-binding.

## Materials and Methods

### Cloning

The recombinant plasmid pET28b expressing PaHigA was a generous gift from Prof. XW Wang lab, SCSIO, CAS, and was constructed as follows (Guo et al., 2019). The full-length of *higA* gene (*PA4674*) was amplified by PCR from *P. aeruginosa* PAO1 genome. The DNA fragment was digested with *Nco*I and *Bam*HI and ligated into the corresponding site of pET28b with a C-terminal His tag (Novagen). The recombinant plasmid was transformed into an *E. coli* BL21(DE3) pLysS expression strain (Invitrogen). Site-directed mutagenesis of *higA* was performed by a PCR-based technique according to the QuickChange site-directed mutagenesis strategy (Stratagene) following the manufacturer’s instructions. The mutant genes were sequenced and found to contain only the desired mutations. The primers used in the study are listed in Supplementary Table S1.

### Protein Expression and Purification

Bacterial cells were grown to mid-log phase (OD_600 nm_ = ∼0.8) in LB media at 37°C in the presence of 50 μg/mL Kanamycin and 100 μg/mL chloroamphenicol. Induction of the culture was then carried out with 0.3 mM isopropyl-1-thio-β-D-galactopyranoside (IPTG) at 20°C. Cells were pelleted after 20 h by centrifugation at 8, 000 rpm for 10 min at 4°C. The cell pellet was resuspended in buffer A [20 mM Tris, 300 mM NaCl, 5% (v/v) glycerol, and 1 mM PMSF, pH 8.0] and lysed by ultrasonification on ice. The cell debris and membranes were pelleted by centrifugation at 15,000 rpm (R20A2 rotor, Hitachi high-speed refrigerated centrifuge R21GIII) for 45 min at 4°C. The soluble C-terminally hexahistidine-tagged PaHigA was purified by affinity chromatography with nickel-nitrilotriacetic acid resin (Bio-Rad). Untagged proteins were removed with buffer A containing 50 mM imidazole. Recombinant PaHigA was then eluted with buffer A containing 250 mM imidazole. The protein was further purified by gel filtration (Superdex 200, GE Healthcare) equilibrated in buffer B [20 mM Tris, 300 mM NaCl, 5% (v/v) glycerol, pH 8.0] using an ÄKTA Purifier System (Amersham).

### Crystallization, Data Collection, Structure Determination and Refinement

The purified PaHigA in complex with the 28-bp or 18-bp DNA was concentrated to ∼6 mg/mL using a Millipore Amicon Ultra apparatus. The initial crystallization conditions were obtained through utilization of several sparse matrix screens (Emerald BioSystems, United States) with the sitting drop vapor diffusion method at room temperature after 2–3 days. Crystal quality was optimized by adjusting the concentration of the precipitant and buffer. The best crystals of PaHigA with 28-bp or 18-bp DNA were both obtained in solution 0.1 M MES (pH 6.5), 35% 3-methyl-1,5-pentane diol (MPD) and 0.2 M lithium sulfate at 20°C.

The diffraction data from a single crystal were collected on the beamline station BL19U of SSRF (Shanghai Synchrotron Radiation Facility) using a Pilatus 6M detector at a wavelength of 0.9788 Å. The total oscillation was 360° with 1° per image and the exposure time was 1 s per image. Before data collection, the crystals were soaked in the reservoir solution supplemented with 20% (v/v) glycerol for a few seconds and then flash-frozen in liquid nitrogen. All the data were processed by XDS (Kabsch, 2010). The initial phases were calculated using the program PHASER with the uncharacterized HigA (PDB ID: 3TRB) as the searching model. The structure was refined with the program Phenix.refine (Adams et al., 2002) and manually corrected in Coot (Emsley et al., 2010). The qualities of the final models were validated with the program MolProbity (Chen et al., 2010). Refinement statistics and model parameters are given in Table 1. The program PyMOL^1^ was used to prepare structural figures.

**TABLE 1:** X-ray data collection and refinement statistics.

Data collection	PaHigA–DNA(28 bp)	PaHigA–DNA(18 bp)
Beamline	SSRF 18U1	SSRF 18U1
Wavelength (Å)	0.9788	0.9788
Space group	*P*2_1_2_1_2_1_	*P*2_1_
Unit-cell parameters	a = 77.8 Å, b = 90.7 Å, c = 91.7 Å, α = β = γ = 90°	a = 57.3 Å, b = 95.6 Å, c = 128.9 Å, α = γ = 90°, β = 96.3
Resolution (Å)	3.14 (3.23–3.14)^a^	2.50 (2.54–2.50)^a^
Number of unique reflections	11506 (807)	47471 (2352)
Completeness (%)	98.3 (94.3)	99.3 (99.2)
Redundancy	6.6 (3.8)	6.2 (5.6)
Mean I/o’ (I)	11.20 (1.77)	20.08 (1.64)
Molecules in asymmetric unit	4	8
*R*_merge_ (%)	10.9 (68.5)	10.5 (86.5)
*R*_meas_ (%)	11.0 (75.4)	11.4 (93.5)
CC_1__/__2_	99.9 (97.8)	100.3 (89.3)
**Structure refinement**		
Reflections used in refinement	11436	47323
Resolution range (Å)	45.35–3.14	47.20–2.50
*R*_work_/*R*_free_ (%)	21.5/28.0	21.8/26.2
Protein atoms	2925	5830
Protein residues	369	733
Waters	0	62
**Average B factor (Å^2^)**		
Protein	75.56	65.27
DNA	79.85	55.62
**Ramachandran plot (%)**		
Most favored	94.2	94.7
Allowed	4.7	3.8
Disallowed	1.1	1.3
**R.m.s. deviations**		
Bond lengths (Å)	0.013	0.010
Bond angles (°)	1.494	1.179

*^*a*^The values in parenthesis means those for the highest resolution shell.*

### Small-Angle X-Ray Scattering (SAXS) and Low Resolution Model Building

Synchrotron SAXS experiments were performed on the BL19U2 station of SSRF, equipped with a PILATUS 1M detector (DECTRIS, Switzerland) (Supplementary Table S2). The scattering was recorded in the range of the momentum transfer 0.018 Å^–1^ < s < 0.321 Å^–1^, where *s* = 4πsinθ/λ, 2θ is the scattering angle, and λ = 1.03 Å is the X-ray wavelength. The solutions were loaded in a flow-through quartz capillary cell with a diameter of 1 mm and a wall thickness of 10 μm, temperature controlled at 22°C. The radiation damage was checked with 20 successive exposures of 1 s. To exclude concentration dependence, three different concentrations, 1 mg/ml, 3 mg/ml, and 5 mg/ml of purified *apo* PaHigA and PaHigA-DNA complex were prepared and measured. All SAXS data were processed with the program package ATSAS (Petoukhov et al., 2012). The scattering of buffers were subtracted from that of the samples, and the forward scattering *I*(0) and the radius of gyration *R*g were derived by the Guinier approximation *I*(*q*) = *I*(0) exp(-*q*^2^*R*_g_^2/3^) for *qR*_g_ < 1.3 using PRIMUS (Konarev et al., 2003). The pair-distance distribution functions, *p(r)* and the maximal dimension of the macromolecule, D_max_ were calculated using indirect Fourier transformation and the program GNOM (Svergun, 1992). To model the structures of *apo* PaHigA or PaHigA-DNA, 10 independent models were generated with the program DAMMIN (Svergun et al., 2001) in fast mode, compared and aligned with SUPCOMB (Kozin and Svergun, 2001), and averaged with DAMAVER (Volkov and Svergun, 2003) to determine common structural features and representative shapes. Theoretical scattering patterns *I(s)* from the available high resolution crystal structures were calculated by a program CRYSOL (Svergun et al., 1995).

### Isothermal Titration Calorimetry (ITC)

Isothermal titration calorimetry was applied to quantitatively determine the binding affinity of PaHigA to the promoter DNA. For the titration experiments, the protein was purified with the same method as above and were dialyzed against the buffer containing 20 mM Tris (pH 8.0), 100 mM NaCl, and 5% (v/v) glycerol for 24 h. DNA was dissolved in the same buffer as above. The ITC experiments were carried out using a high-sensitivity iTC-200 microcalorimeter from Microcal (GE Healthcare) at 20°C using 400 μM DNA in the injector while 100 μM PaHigA in the sample cell. All samples were thoroughly degassed and then centrifuged to get rid of precipitates. Injection volumes of 2 μL per injection were used for the different experiments and for every experiment, the heat of dilution for each ligand was measured and subtracted from the calorimetric titration experimental runs for the protein. Consecutive injections were separated by 2 min to allow the peak to return to the baseline. Raw heat data obtained for the ITCs were integrated using the MicroCal-Origin 7.0 software package. The integrated data were analyzed using a trimolecular reaction model describing a cooperative binding of PaHigA to the promoter DNA:

(PaHigA_2_ + 1/2 DNA → 1/2 PaHigA_2_-DNA, the subscript “2” means PaHigA homo-dimer).

The model-adjusted parameters Δ*G*_A_ (free energy of association per mole of ligand), Δ*H*_A_ (standard enthalpies of association per mole of ligand) and equilibrium association constants *K*_A_ were calculated by the method used for the DNA-binding of GraA described recently (Talavera et al., 2019).

### Gel Electrophoresis Mobility Shift Assays (EMSA)

The DNA fragments were chemically synthesized by Beijing AuGCT Biotechnology Co. Ltd., China. DNA samples (2 μM final concentration) were annealed in the buffer containing 10 mM NaCl, 20 mM Tris (pH 8.0). PaHigA-DNA complexes were prepared by adding PaHigA at 0, 2, 4, 6, 8, and 10 μM final concentration and incubating for 30 min at room temperature. For each sample, free DNA and complexes were separated on a 5% acrylamide native gel run for 40 min at 80 V at 20°C and visualized by Ethidium bromide (Thermo Fisher Scientific, United States) staining.

### Accession Numbers

The atomic coordinates and structure factors of PaHigA in complex with the 28-bp and 18-bp DNA have been deposited into the RCSB PDB with the accession codes 6JPI and 6LB3, respectively. The SAXS data of *apo* PaHigA and PaHigA-28-bp DNA complex have been deposited into SASBDB with the accession code SASDF85 and SASDF95, respectively.

## Results

### PaHigA Can Directly Bind to the Promoter Region of *higBA* Operon Containing Two Palindromes

**FIGURE 1:**
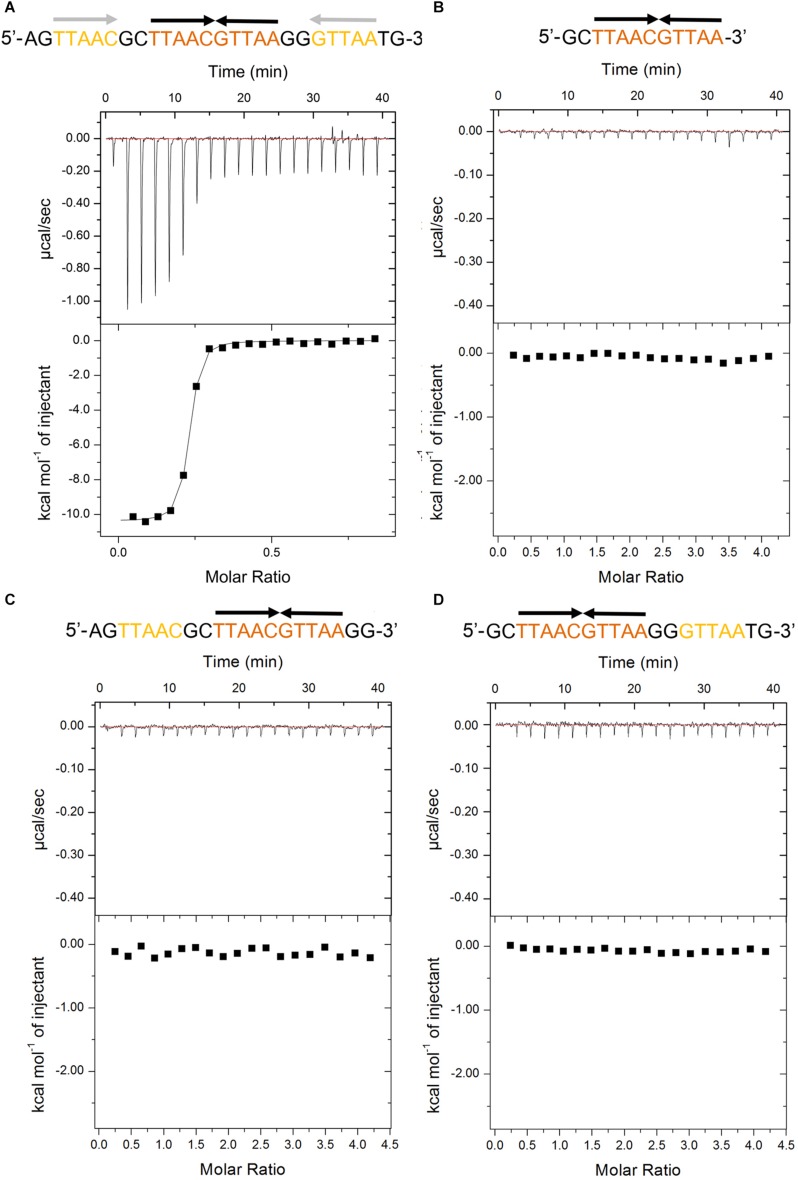
Thermodynamic analyses of PaHigA to the DNA fragments derived from the promoter region of *higBA* promoter in *P. aeruginosa* PAO1. These fragments include containing two identical palindromes **(A)**, central palindrome only **(B)**, central palindrome with either upstream **(C)** or downstream semi-palindromic regions palindrome **(D)**, respectively. The central and distal palindromic sequences of the DNA are shown in orange and yellow, respectively, and the other regions were shown in gray.

The recent promoter analysis identified two identical palindromic sequences (5′-TTAAC GTTAA-3′) in the promoter region of *higBA* operon, which are composed of one central site and two half distal sites (Guo et al., 2019) (Figure 1A). To test if PaHigA can bind directly to DNA in this region, we generated a 28-bp DNA duplex covering two palindromic sequences derived from the *PaHigBA* promoter region (referred to P*_*higBA*_*), and characterized their interaction isothermal titration calorimetry (ITC) experiments (Figure 1A).

PaHigA shows remarkable binding to the operator DNA fragment (*K*_A_ = 7.5 × 10^13^ ± 8.1 × 10^10^ M^–2^, ΔH = −1.04 × 10^4^ M^–1^ ± 74.78 cal/mol), and the apparent binding affinity is similar to that of antitoxin GraA (PaHigA homolog) to its promoter DNA (*K*_A_ = 3.0 × 10^13^ M^–2^) (Talavera et al., 2019). Moreover, to study the roles of the two palindromic sequences in DNA-binding, we also generated the DNA fragments lacking either upstream or downstream semi-palindromic regions (Figures 1C,D) or lacking both regions (Figure 1B), respectively. Unexpectedly, all these variants didn’t show detectable binding to PaHigA by ITC (Figures 1B–D). The results suggested both palindromes are essential for PaHigA-binding. Meanwhile, the size-exclusion chromatography also showed a new, monodisperse peak with reversed A_260_/_A__280_ ratio in the presence of the 28-bp DNA that is eluted earlier than the isolated *apo* PaHigA (Supplementary Figure S1). Together, these experiments demonstrate that PaHigA can form a stable complex with the 28-bp DNA duplex containing the intact palindromic region, which is used for the following crystallography and DNA-binding studies.

### Two PaHigA Dimers Bind to PaHigBA Promoter DNA Symmetrically

To elucidate the molecular mechanism of DNA recognition by PaHigA, we determined the crystal structures of PaHigA in complex with the 28-bp DNA duplex. The structure was determined by molecular replacement using the uncharacterized HigA from *Coxiella burnetii* (PDB ID: 3TRB), and were refined to a final *R*/*R*_free_ factor of 0.21/0.28 at 3.14 Å (Table 1). The asymmetric unit contains one DNA fragments binding four PaHigA molecules, which form two homo-dimers. Structural superposition of the two dimers showed their overall conformations are highly similar (r.m.s.d. 1.2 Å for 182 Cα atoms). The electron density for the DNA fragment was well defined in the initial difference maps and the whole 28-bp DNA model can be unambiguously built (Supplementary Figure S2).

**FIGURE 2:**
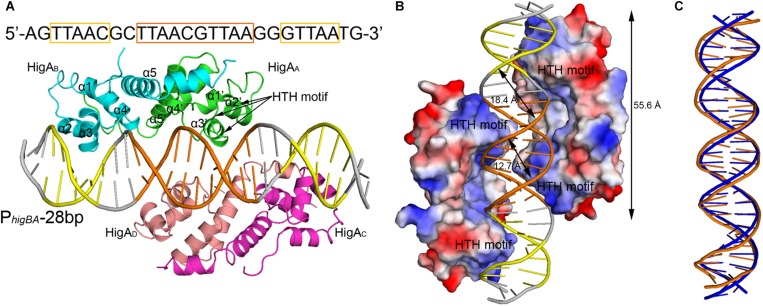
Overview of PaHigA-P*_*higBA*_* complex structure. **(A)** Overall crystal structure of PaHigA in complex with a 28-bp DNA duplex derived from P*_*higBA*_* at 3.14 Å resolution. The four protein protomers are labeled using the subscripts A–D in green, cyan, magenta and pink, respectively. The central and two semi-palindromic sequences of the DNA are shown in orange and yellow, respectively, and the other regions were shown in gray. **(B)** The molecular surface representation of the complex with a 90° rotation from **(A)** (blue, +7.9 KT; red, –7.9 KT), colored by its local electrostatic potential. **(C)** Conformational comparison of the 28-bp DNA duplex (orange) and canonical B-DNA (blue).

The two PaHigA dimers bind to the B-form DNA duplex symmetrically, where each dimer bound on the opposite sides (Figures 2A,B). The average width of the major groove and minor groove of the promoter DNA is 18.4 and 12.7 Å, respectively, whereas they are 17.2 and 11.7 Å in the ideal B-form DNA, respectively, which are calculated by the w3DNA web server (Supplementary Figure S3) (Li et al., 2019). The two identical palindromic sequences are essentially encircled by the two PaHigA dimers simultaneously. The total buried surface area between the two PaHigA dimers and the DNA is ∼2340 Å^2^, is almost evenly distributed in each protomer. The helix-turn-helix family (HTH) motifs from each PaHigA dimer insert into the major grooves of the promoter DNA, which is composed of a central palindrome (TTAAC GTTAA, in orange) and two semi-palindromic regions (in yellow). Moreover, the predominantly positive charge of the HTH motifs complements well with the negative DNA phosphate backbone to induce favorable binding (Figure 2B). The DNA fragment adopts a canonical B-DNA conformation without significant bending (Figure 2C). The stoichiometry of PaHigA-DNA is unique in known antitoxin-promoter DNA structures, in which an inverted repeat in the operon region is usually bound by an antitoxin dimer.

**FIGURE 3:**
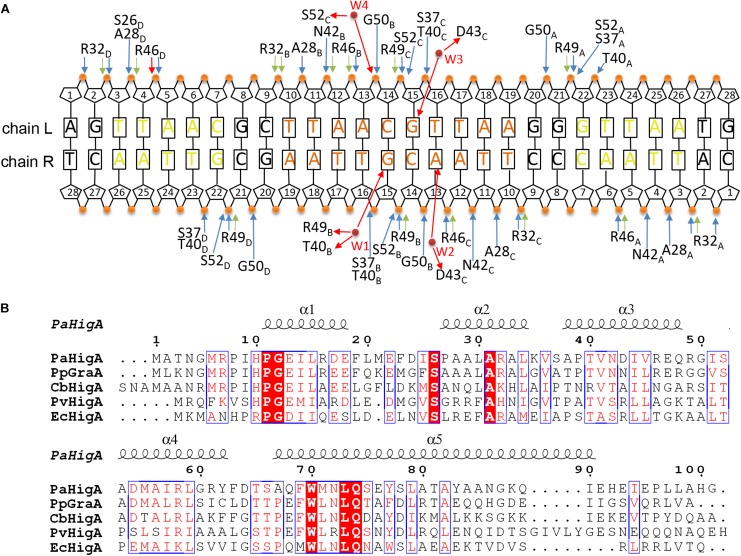
Contacts analysis of PaHigA and DNA in the complex structure. **(A)** Schematic overview of the interactions between PaHigA and DNA. The direct interactions involve the hydrogen bonds (blue) and electrostatic interactions (green) to phosphate groups (orange circles). The indirect interactions (red) are mediated by four water molecules (W1–W4, red circles) to the base pairs (W2–W4) or phosphate groups (W1). It should be noted the water-mediated interactions are only found in the structure of PaHigA in complex with a 18-bp DNA duplex at 2.50 Å resolution. **(B)** Structure-based sequence alignment of PaHigA with its representative homologs performed using clustal X (version 1.81) and ESPript 3. They include GraA from *P. putida* (PpGraA) and HigA from *Coxiella burnetii* (CbPaHigA), *Proteus vulgaris* (PvPaHigA) and *E. coli* (EcPaHigA).

Analysis of the complex reveals the four HTH motifs from the two dimers insert into the major grooves of the DNA symmetrically. The DNA-contacting residues in each protomer are highly similar (Figure 3A). Moreover, only several residues, such as S26, R32 and T40, are conserved among HigA homologs from different species (Figure 3B). Interestingly, all the direct contacts, including the hydrogen bonds (H-bonds) and electrostatic interactions, are mediated by the DNA phosphate backbone.

### PaHigA Can Form a Compact Homo-Dimer

PaHigA is composed of a compact five α-helical bundle medicating a hydrophobic core (Figure 2A). The residues Met1-Gly5 and Leu98-Gly101 were not observed in the electron density map and not included in the current model because of their flexibility. Two PaHigA monomers can form a compact homo-dimer by making extensive contacts (hydrogen bonds and hydrophobic interactions). The dimerization interface is formed by the helices α4 and extended helices α5 (Figure 2A). The buried surface area in the dimer interface is 1,458 Å^2^, which is 22.4% of the total surface area per monomer (11,533 Å^2^). The structure of the PaHigA dimer in the context of PaHigA-DNA complex is very similar to that of *apo* HigA from *C. burnetii* (PDB ID: 3TRB, with Z-score 16.3 and r.m.s.d. 0.9 Å for 92 Cα atoms) and from *E. coli* CFT073 (PDB ID: 2ICP, with *Z*-score 12.6 and r.m.s.d. 1.2 Å for 87 Cα atoms). Moreover, PaHigA also showed remarkable similarities to HigB-binding HigA from *Proteus vulgaris* (PDB ID: 4MCX, with *Z*-score 13.4 and r.m.s.d. 2.1 Å for 92 Cα atoms) and GraT-binding GraA from *Pseudomonas putida* (PDB ID: 6F8S, with Z-score 15.4 and r.m.s.d. 1.5 Å for 89 Cα atoms) (Supplementary Figure S4). This suggests PaHigA may not undergo remarkable conformational changes upon DNA-binding or HigB-binding.

The helices α2 and α3 form a characteristic HTH motif that likely binds its own or other DNA promoters to repress TA transcription. This motif has been reported in several HigA homologs and the DNA-binding xenobiotic response element XRE-HTH family members (Luscombe et al., 2000). However, the HTH motif is located in the N-terminus in the N-terminus of PaHigA, PvHigA and EcHigA (strain CFT073), where in the C-terminus of EcHigA (strain K12) and *Vibrio cholerae* HigA2 (VcHigA2). Moreover, these HigA homologs are fully structured in both the complex and the *apo* form (except the N-terminal region of VcHigA2 is intrinsically disordered), contrary to most antitoxins with overall or partial flexible conformations (Loris and Garcia-Pino, 2014; Page and Peti, 2016).

### PaHigA Binds to the Promoter DNA by a Cooperative Dimers

**FIGURE 4:**
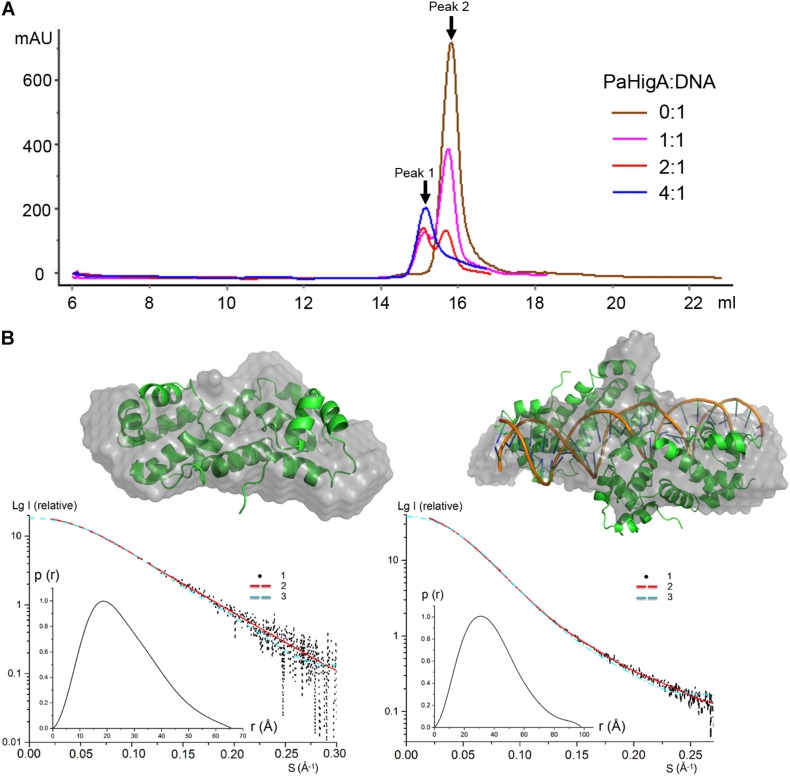
PaHigA binds to the promoter DNA by two cooperative dimers. **(A)** Analysis of binding of PaHigA-DNA complexes formed by different PaHigA:DNA molar ratios using gel filtration chromatography (Superdex^TM^ 200 10/300 GL). Peak 1 and 2 represent a stable protein-DNA complex and excess DNA, respectively. **(B)** Solution conformations of *apo* PaHigA (Left) and PaHigA-DNA complex (Right, the samples from Peak 1 above) by SAXS analysis. Curve 1-SAXS experimental data; Curve 2: scattering patterns computed from the DAMMIN model; Curve 3-scattering patterns computed from the atom model by the program CRYSOL; Inserts: lower left, P(r) function; upper right, DAMMIN models overlapping with the crystal structures.

To investigate whether the of two PaHigA dimers bind to the promoter DNA in a cooperative way, different PaHigA: DNA ratios were designed to study their binding characteristics by a series of analytical size exclusion chromatography (SEC) experiments. The results showed that in spite of different stoichiometry of the initial mixture (PaHigA: DNA = 1:1, 2:1, and 4:1), there are always two major peaks (Peak 1 and 2), representing a stable protein-DNA complex and excess DNA, respectively (Figure 4A). The elution volume of Peak 1 (∼15.1 ml) in each stoichiometry is very similar to that of purified proteins used for crystallization (Supplementary Figure S1).

The oligomeric state of the protein-DNA samples from Peak 1 (PaHigA: DNA = 1:1) was further studied by SAXS in solution (Figure 4B, Supplementary Figure S5, and Supplementary Table S1). The results show that the experimental SAXS curves are in agreement with the *apo* PaHigA dimer theoretical curve (χ^2^ = 1.064) (Supplementary Table S2). Moreover, the calculated molecular mass (MS) from the SAXS data (∼22.1 kDa) also indicated it is a homo-dimer that is similar to its homologs from other species. Meanwhile, comparison of the theoretical scattering patterns from the crystal structure of PaHigA-DNA complex with the experimental SAXS profile showed that the model curve differs considerably calculated by CRYSOL (χ^2^ = 6.29). However, considering that the MS of the DNA used is 18.7 kDa and that PaHigA-His forms a dimer of ∼25 kDa, the calculated MS from the SAXS data (73.8 kDa) is compatible with that from the crystal structure (∼69 kDa) (Supplementary Table S2). The crystal structure can be roughly fit into the SAXS-derived low resolution envelope (Figure 4B). Moreover, the calculated MS of Peak 1 from in the stoichiometry of 2:1 and 4:1 by SAXS analysis are also ∼75 kDa (data not shown). All these data suggest two PaHigA dimers can bind to the promoter DNA in a cooperative manner to form a stable complex under different protein-DNA stoichiometry.

### Three Water Molecules Mediated Nucleobase-Specific Interactions With PaHigA

**FIGURE 5:**
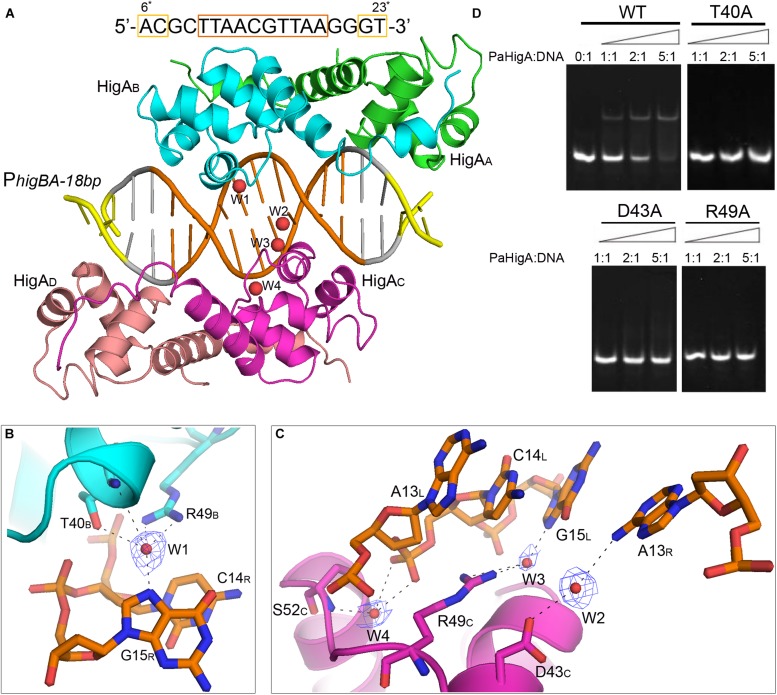
Water-mediated interactions between PaHigA and DNA. **(A)** Overall crystal structure of PaHigA in complex with a 18-bp DNA duplex derived from P*_*higBA*_* at 2.50 Å resolution. The colorings are the same as those in Figure 2A. Four water molecules (W1–W4) are incorporated into the protein-DNA interface. The water molecules were shown as red spheres. It should be noted the DNA fragment is numbered from 6^∗^ to 23^∗^ that is consistent with the DNA numbering in the 28-bp DNA duplex. **(B,C)** Water-mediated base pair interactions between PaHigA monomers and DNA. The hydrogen bonds mediated by water molecules are represented by dotted lines. Electron density maps (2Fo _ Fc) of the water molecules are contoured at the 1.2 σ (blue mesh). **(D)** EMSA assays for the DNA binding capacity of PaHigA mutants. The DNA substrates (2 μM) were co-incubated with increasing amount (0–10 μM) of PaHigA and loaded onto 5% polyacrylamide/bis (37.5:1) gels and run at a constant voltage of 80 V.

Because no direct contacts between DNA bases and PaHigA, we tried to find whether there are solvent-mediated nucleobase-specific interactions in this structure. However, no water or other solvent molecules can be modeled at 3.14 Å resolution. Then we tried the co-crystallization of PaHigA with several DNA fragments with various length to improve the structure resolution. The structure of PaHigA in complex with a 18-bp DNA covering the central palindromic region and partial semi-palindromic region was solved at 2.50 Å resolution (Table 1, Figure 5A, and Supplementary Figure S2). The overall architectures of PaHigA in complex with the 18-bp and 28-bp DNA are highly similar (with r.m.s.d. 0.63 Å for 369 Cα atoms, Supplementary Figure S6).

In the 2.50 Å resolution structure, four water molecules (W1-W4) can be modeled (with the B-factor 47.32, 49.04, 53.3, and 51.04, respectively) on the DNA-binding interface in the palindromic region. Three water molecules (W1-W3) mediated hydrogen bonds between PaHigA (Thr40, Asp43 and Arg49) and DNA base pairs, and one water molecule (W4) mediates the hydrogen bonds between protein (Ser52) and the DNA backbone (Figures 5B,C). The side chains of both Thr40 and Arg49 in monomer B participate in water-mediated interactions with the N7 atom of guanine 15 (chain R). Asp43 and Arg49 in monomer C participate in water-mediated interactions with the N6 atom of adenine 13 (chain R) and the N7 atom of guanine 15 (chain L), respectively. Moreover, the side chains of Thr40 and Arg49 make multiple hydrogen bonds to the phosphate backbone of the promoter DNA (Figure 3A).

In order to evaluate the role of these residues in DNA-binding, they were mutated to alanine to test their DNA-binding abilities by electrophoretic mobility shift assay (EMSA) experiments. The EMSA results showed that wild-type PaHigA bound and shifted the DNA fragment expectedly. When an increasing amount (2–10 μM) of protein is co-incubated with DNA, no obvious shifted bands are observed for all these three mutants compared to the wild-type (Figure 5D). The results showed disruption of the hydrogen bonds mediated by water molecules will abolish the DNA-binding activities. These data indicate that the three residues are essential for the specific interaction between PaHigA and its cognate promoter DNA mediated by water molecules.

## Discussion

Four characteristic DNA-binding motifs, including HTH (Brown et al., 2009; Schumacher et al., 2009; Schureck et al., 2014), RHH (ribbon-helix-helix) (De Jonge et al., 2009; Bøggild et al., 2012), Phd/YefM (Garcia-Pino et al., 2010), and SpoVT/AbrB (Dienemann et al., 2011), have been reported in various antitoxin structures mediating diversified repressor mechanisms. In this study, using biochemical and structural analyses, we provide a detailed mechanistic basis into the regulation mechanism of HigBA operon transcription that is associated with *P. aeruginosa* pathogenicity. Such recognition mechanism that the promoter DNA-binding by a cooperation of two antitoxin dimers is rare in TA operon transcriptions, which the operon region is usually bound by one antitoxin dimer (Loris and Garcia-Pino, 2014; Page and Peti, 2016).

**FIGURE 6:**
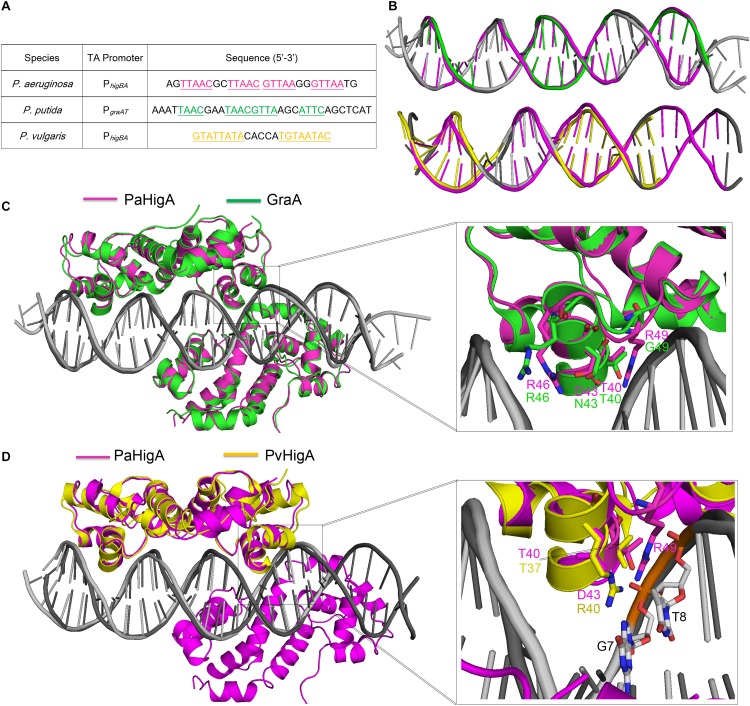
Structural comparison of PaHigA-DNA with two recently reported HigA family members. **(A)** The promoter sequences of PaHigA, *P. putida* GraA and *P. vulgaris* HigA (PvHigA). The palindromic sequences are underlined and highlighted in magenta, green and yellow, respectively. **(B)** Comparison of the DNA conformations of P*_*higBA*_* with those of the other two promoters. **(C,D)** Structural superposition of PaHigA-DNA (in magenta) with *P. putida* GraA-DNA (PDB ID: 6FIX, in green) and *P. vulgaris* HigA-DNA (PDB ID: 6CHV, in yellow) in the overall view (Left) and close-up view of the DNA-binding interface (Right).

Until recently, the structures of *P. putida* GraA-DNA (56% sequence identity with PaHigA) and *P. vulgaris* HigA-DNA (22% sequence identity with PaHigA) demonstrated the different repressor mechanisms of HigBA family members (Schureck et al., 2019; Talavera et al., 2019). Overall, no significant bending is observed and the promoter DNA does not deviate much from the ideal B-DNA conformation in all these three structures (Figures 6A,B). However, there is a bend of ∼55° and ∼70° of the promoter DNA that is induced by the shorter distances between the binding helices in HipB and MqsA harboring a HTH motif, respectively (Brown et al., 2011; Wen et al., 2014; Schumacher et al., 2015). In GraA-DNA structure, two GraA dimers bind cooperatively at opposite sides of the promoter sequence containing two palindromes like that observed in PaHigA-DNA structure (Talavera et al., 2019). However, the palindrome sequence in the distal site of *graAT* promoter DNA is not strictly palindromic (TAAC and ATTC, Figure 6A). Most of the residues (such as R46) involved in DNA backbone-binding are conserved in PaHigA and GraA (Figure 3B). However, R49 and D43 that play an essential role in water-mediated specific DNA base recognition in PaHigA are not conserved in GraA (corresponding to G49 and N43, respectively, Figure 6C). The structure of *P. vulgaris* HigA-DNA complex showed the promoter DNA with a single inverted repeat was recognized by only one PvHigA dimer (Figure 6D) (Schureck et al., 2019). PvHigA recognizes its promoter by the side chain of the conserved arginine residue (R40) in HTH motif. R40 makes both nucleobase-specific interactions with a guanosine and pi-cation stacking interactions with an adjacent thymine in recognition of an YpG dinucleotide (Figure 6D). The key residues R40 and T37 in PvHigA correspond to D43 and T40 in PaHigA, respectively (Figures 3B, 6D), which also play essential roles in specific DNA-binding. Overall, PaHigA may adopt a similar promoter DNA-binding mechanism by two cooperative dimers like GraA, whereas distinct from that of PvHigA.

In both PaHigA-28bp DNA structure (3.14 Å) and GraA-DNA structure (3.80 Å), all the direct contacts (hydrogen bonds) are mediated by the DNA phosphate backbone to the protein, and no water molecule can be modeled under the relatively lower resolution. In the following 2.50-Å PaHigA-18 bp DNA structure, several disordered water molecules were modeled on the DNA-binding interface and were found play essential roles for the specific interaction between PaHigA and its cognate promoter DNA. Analysis of PaHigA-DNA structure showed all these waters are located in the DNA major groove. This may be attributed by the relatively slighter insertion of the HTH motifs into the major groove. The incompact binding may leave suitable space to accommodate water molecules on the DNA-binding interface, which in turn mediate the specific recognition DNA bases by HigA. The water-mediated interactions have been found to play important roles in DNA-binding (Otwinowski et al., 1988; Kalodimos et al., 2004; Yang et al., 2013). A typical example is the structure of a broad regulator Ms6564-DNA in *Mycobacterium smegmatis*, in which the DNA-binding motif doesn’t insert deeply into the DNA major groves compared to that of QacR (Ms6564 homolog). Eleven water molecules are involved in bridging the protein-DNA interaction, in which seven water molecules bridge the contacts between Ms6564 and DNA base pairs and four mediate hydrogen bonds between protein and the DNA backbone. The incompact binding was suggested to allow Ms6564 to easily slide on the genome and efficiently recognize the specific motif. Considering the potential regulation of multiple virulence factors by PaHigA, the incompact binding may also be required to efficiently recognize the palindromic specific motif in the genome.

The recent work showed PaHigA can specifically bind to the promoter of the virulence gene *mvfR* containing a palindromic sequence identical to the central palindrome in *higBA* operon (Guo et al., 2019). Moreover, a HigA-like palindrome (5′-TTGAC GTTAA-3′, compared to 5′-TTAAC GTTAA-3′ in *higBA* and *mvfR* operons) was identified in the promoter of the *pel* operon, which is responsible for Pel polysaccharide synthesis (Ryder et al., 2007). In our structure, this palindromic region is located in the center of the promoter DNA and is completely encircled by two PaHigA dimers. The central location suggests this palindrome may function as the core element in promoter DNA binding. Our EMSA experiments showed PaHigA can bind to the DNA fragments (30 bp) including the palindromic sequences that are derived from the promoters of *mvfR* (P*_*mvfR*_*) and *pelA* (P*_*pelA*_*), respectively (Supplementary Figure S7). Obvious shifted bands are observed for P*_*mvfR*_* and P*_*pelA*_* at higher protein:DNA ratios compared to that of P*_*higBA*_* (Figure 5D). The results suggest the central palindrome are essential and specific for PaHigA binding. On the other hand, considering the notable binding of P*_*mvfR*_* and P*_*pelA*_* in the absence of the upstream and downstream semi-palindrome in P*_*higBA*_*, we may conclude the two semi-palindromes are not required for PaHigA binding. Meanwhile, PaHigA shows no detectable binding to the central palindrome only (Figure 1), indicating it is necessary but insufficient for PaHigA binding. Indeed, our structures showed the all four protomers bind to the DNA fragment by numerous interactions (Figure 3A), and the DNA truncations may significantly affect the protein-binding and destabilize the protein-DNA complex. All these data suggest the DNA with sufficient length in the upstream and downstream of the central palindrome are therefore required for two PaHigA dimers binding simultaneously, although such DNA sequences are non-specific. The recent DNA-bindings studies of GraA (PaHigA homolog) also showed the central palindrome is a major binding motif within the promoter, whereas the two half distal palindrome sites provide non-specific but necessary contacts for DNA-binding in GraA (Talavera et al., 2019).

In summary, our structure-function studies demonstrate the novel binding manner of promoter DNA by a cooperation of PaHigA dimers that differs from most TA systems. The specific recognition is achieved by water-mediated the interactions between the bases of palindromic sequences and PaHigA. Our findings may also help to understand the cross-regulation the *mvfR* operon and *pel* operon that are associated pyocyanin synthesis and biofilm formation in *P. aeruginosa*.

## Data Availability Statement

The atomic coordinates and structure factors of PaHigA-DNA complex have been deposited into the RCSB PDB with the accession code 6JPI. The SAXS data of *apo* PaHigA and PaHigA-DNA complex have been deposited into SASBDB with the accession code SASDF85 and SASDF95, respectively.

## Author Contributions

HZ and YD conceived and designed the experiments. YL, ZGa, and GL performed the experiments. HZ, YL, ZGe, GL, and YD analyzed the data. HZ, YL, and YD wrote the manuscript.

## Conflict of Interest

The authors declare that the research was conducted in the absence of any commercial or financial relationships that could be construed as a potential conflict of interest.

## Footnotes

1 http://www.pymol.sourceforge.net/
