# Supplementary material for: Structural Insights Into the Transcriptional Regulation of HigBA Toxin–Antitoxin System by Antitoxin HigA in Pseudomonas aeruginosa
Source: Front Microbiol. 2020 Jan 22;10:3158. doi: 10.3389/fmicb.2019.03158 (PMC6987408; doi:10.3389/fmicb.2019.03158)
Supplement: Supplementary file 1 [file Data_Sheet_1.docx]

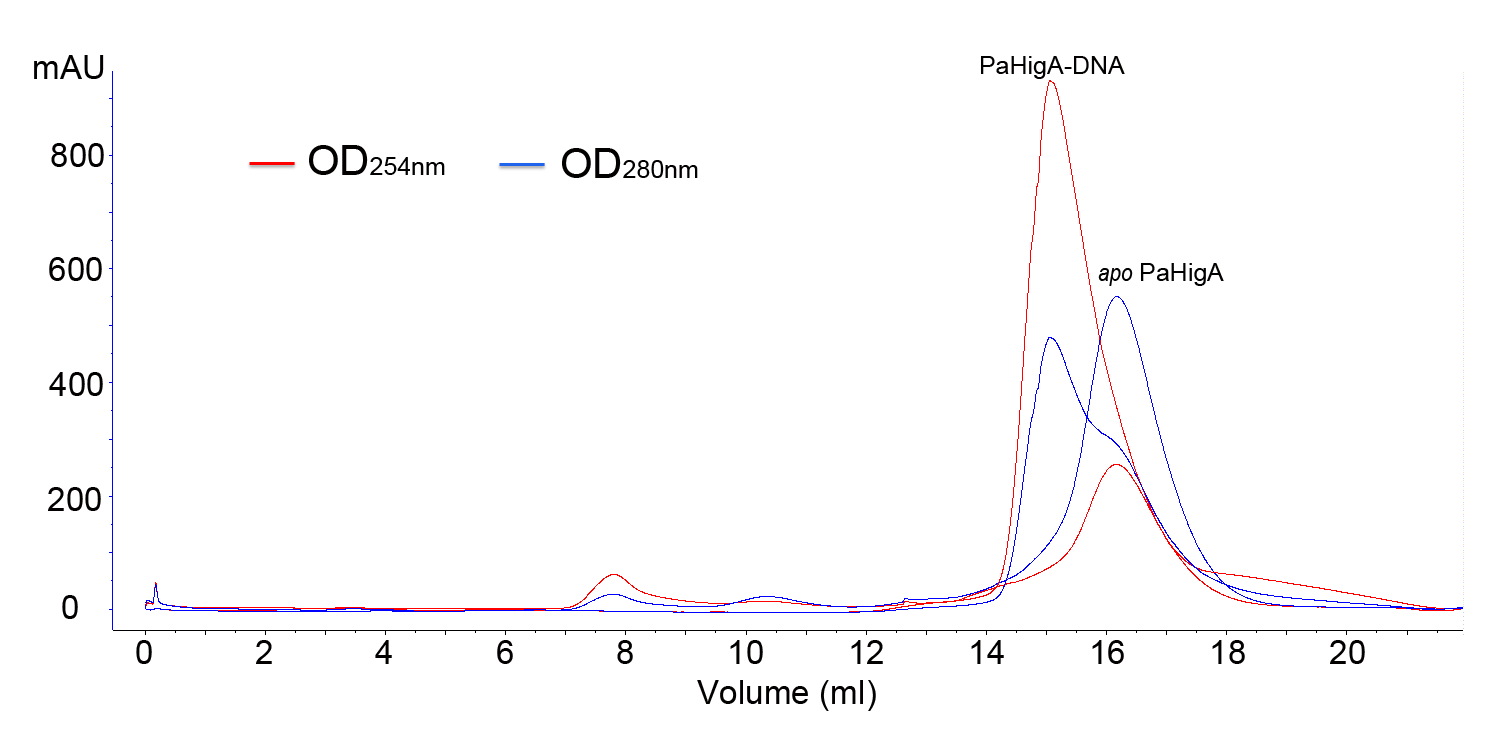


**Figure S1. Analysis of binding of a 28-bp DNA duplex derived from *higBA* promoter by size-exclusion chromatography.** Purified *apo* PaHigA and PaHigA-DNA complex eluted from gel filtration chromatogram (Superdex^TM^ 200 10/300 GL) at 16.2 and 15.1 ml, respectively.


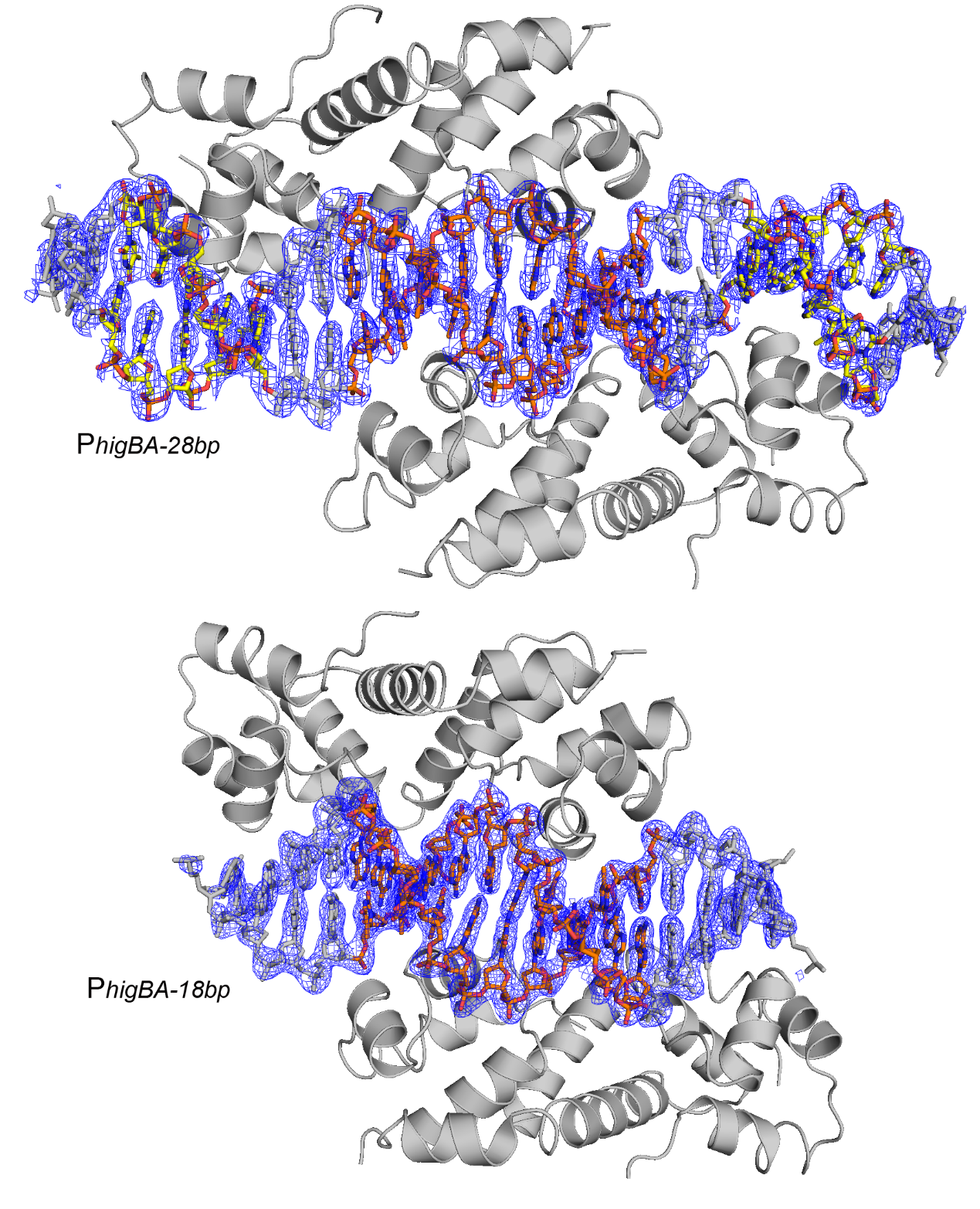


**Figure S2. 2Fo-Fc electron density map (blue) of the 28-bp and 18-bp DNA duplex contoured at 1.5 σ level.**


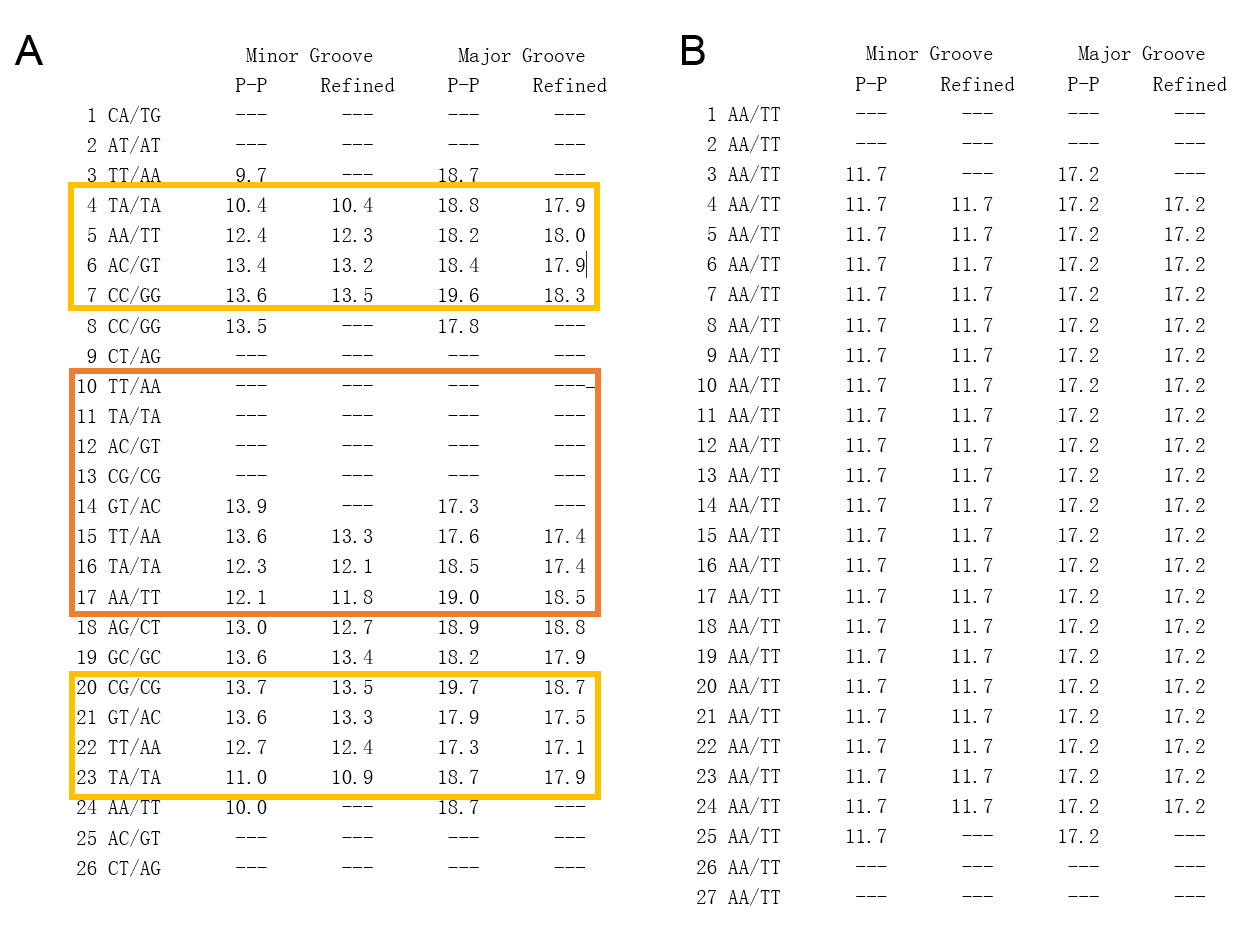


**Figure S3.** **The width of the major groove and minor groove of the promoter DNA (A) and the ideal B-form DNA calculated by the w3DNA web server (http://web.x3dna.org/).** Minor and major groove widths use direct P-P distances and refined P-P distances which take into account the directions of the sugar-phosphate backbones (Subtract 5.8 Angstrom from the values to take account of the vdw radii of the phosphate groups, and for comparison with FreeHelix and Curves.)


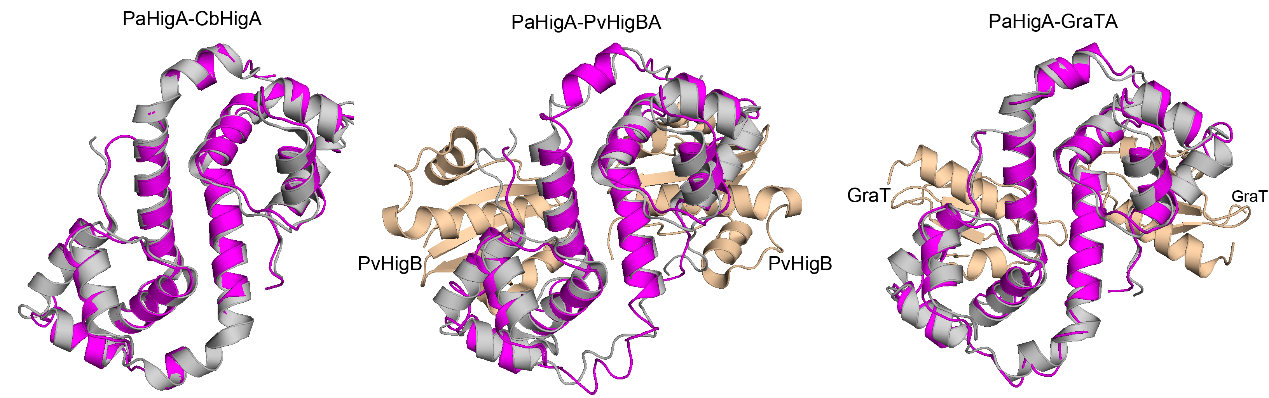


**Figure S4. Structural superimposition of DNA-binding PaHigA dimer with its homologs in *apo* form or toxin-binding form.** PaHigA is shown in magenta. Apo CbHigA (PDB ID: 3TRB, with Z-score 16.3 and r.m.s.d. 0.9 Å for 92 Cα atoms), PvHigA (PDB ID: 4MCX, with Z-score 13.4 and r.m.s.d. 2.1 Å for 92 Cα atoms) and GraA (PDB ID: 6F8S, with Z-score 15.4 and r.m.s.d. 1.5 Å for 89 Cα atoms) are shown in gray, and the corresponding toxins are shown in wheat.


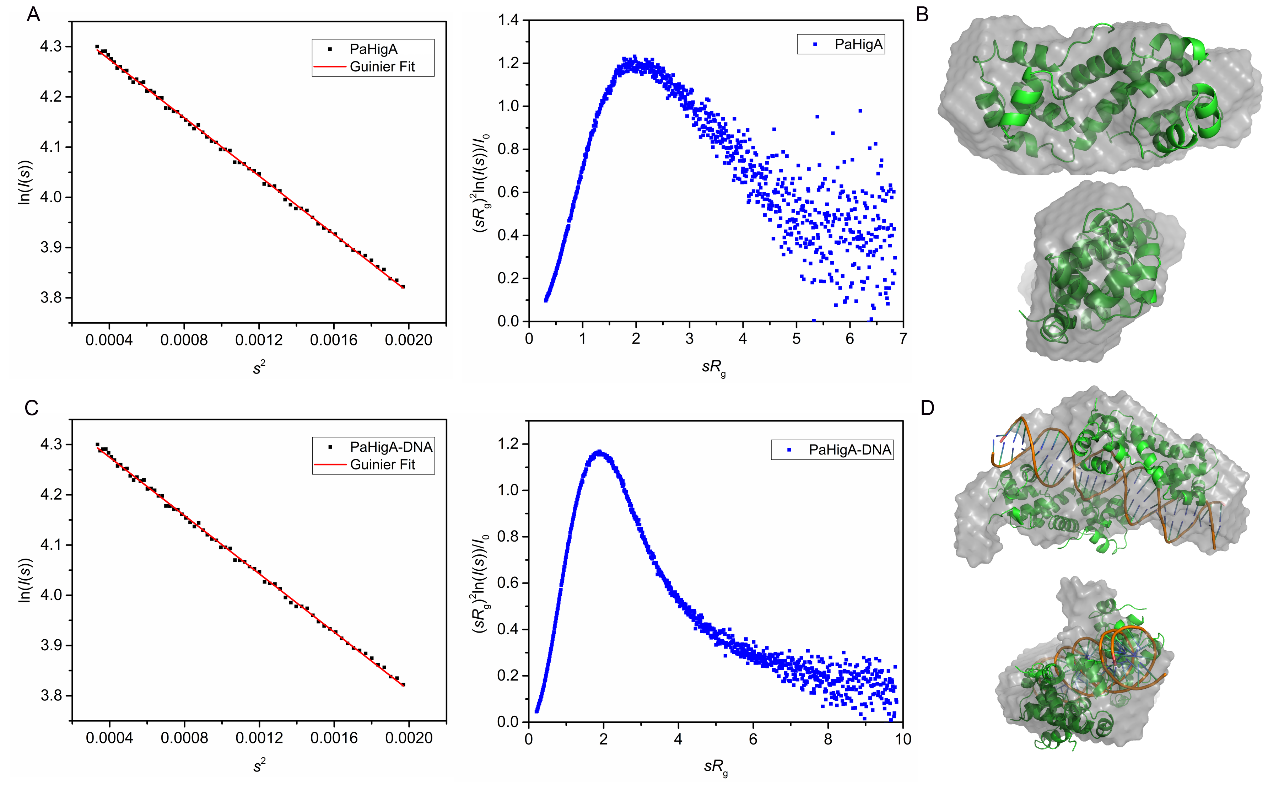


**Figure S5.** Guinier plot and Kratky plot analysis of *apo* PaHigA (A-B) and PaHigA-DNA complex (C-D) by SAXS.


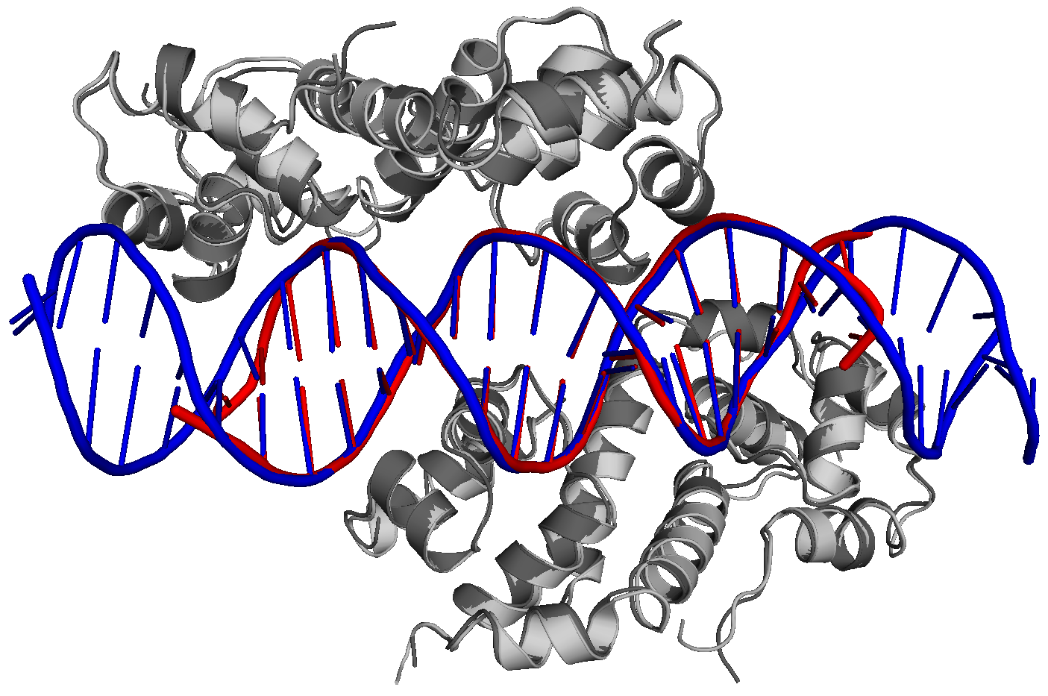


**Figure S6. Structural superposition of the structures of** **PaHigA in complex with the 28-bp and 18-bp DNA duplex.** The proteins in the two structures are shown in light gray and dark gray, respectively. The two DNA fragments are highlighted in blue and red, respectively.


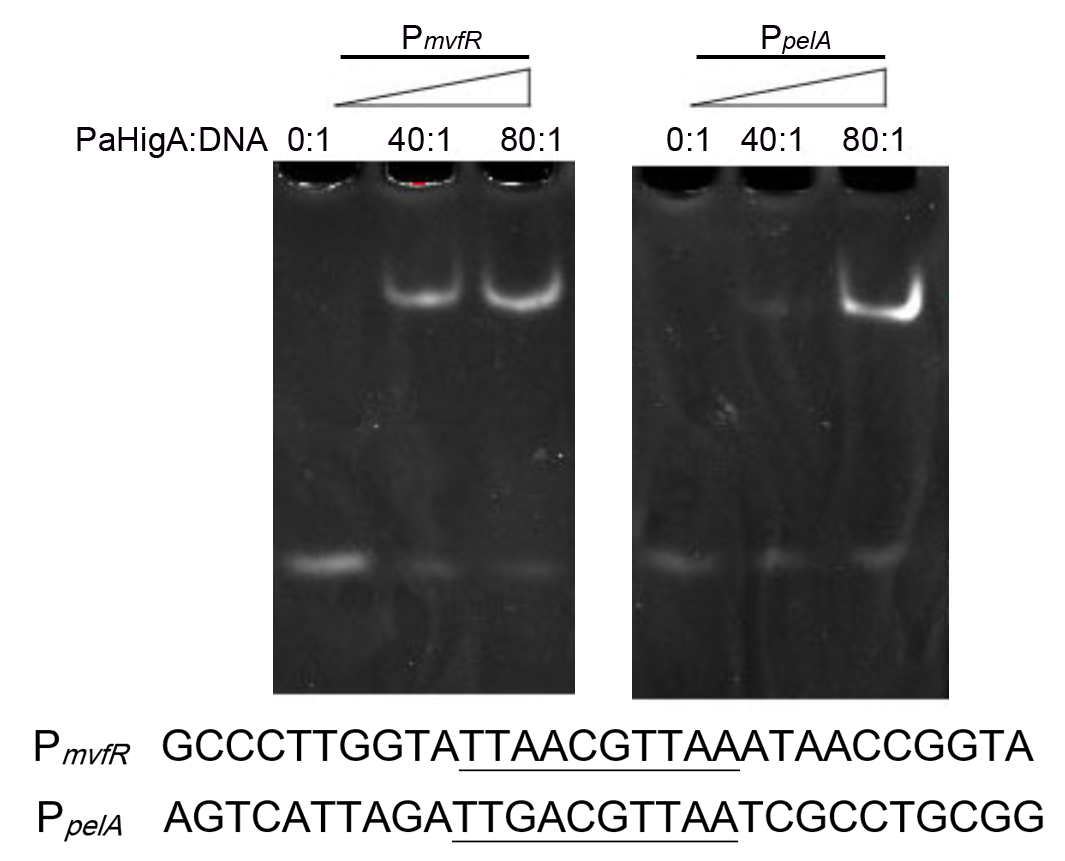


**Figure S7.** EMSA assays for the DNA binding capacity of PaHigA to the DNA fragments derived from the promoters of *mvfR* (P*_mvfR_*) and *pelA* (P*_pelA_*). The palindromic sequences are underlined.

**Table S1.** The list of PaHigA (wild-type and mutants) primers in this study. The mutated nucleic acids are shown in lower case. Restriction enzyme sites are underlined. F indicates forward primer and R indicates reverse primer.

| **primer** | **sequences** | |
| --- | --- | --- |
|  | | |
| Wild-type | F | 5’- CTAGCCATGGGCATGGCTACCAATGGTATGCGCC -3’ |
|  | R | 5’-CGCGGATCCCTAGTGGTGGTGGTGGTGGTGTCCGT GAGCAAGCAGCGGC-3’ |
| R49A | F | 5’-ATCGTTCGTGAGCAG gc TGGTATCT -3’ |
|  | R | 5’-gc CTGCTCACGAACGATATCGTTCA -3’ |
| T40A | F | 5’-AAAGTCTCCGCTCCG g CAGTGAAC -3’ |
|  | R | 5’-c CGGAGCGGAGACTTTCAAAGCGC -3’ |
| D43A | F | 5’-CTCCGACAGTGAACG c TATCGTTCGTG -3’ |
|  | R | 5’-gCGTTCACTGTCGGAGCGGAGACTTTC’ |

**Table S2. Statistics on SAXS data collection, analysis and modeling.**

| Data Collection Parameters | | | |
| --- | --- | --- | --- |
| Instrument | | BL19U2 beamline, equipped with a Pilatus 1M Detector | |
| Beam geometry | | Scatterless Slits | |
| Wavelength (Å) | | 1.033 | |
| qrange (Å^-1^) | | 0.01-0.31 | |
| Temperature (K) | | 295 | |
| Exposure time (s) | | 1s per frame | |
| Protein concentration (mg/ml) | | 1, 3 and 5 | |
| **Protein samples** | | ***apo* PaHigA** | **PaHigA-DNA complex** |
| Structural Parameters | | | |
| From Guinier fit | I(0) (cm^-1^) | 18.77±0.04 | 80.36±0.17 |
|  | Rg(Å) | 19.66±0.07 | 29.35±0.08 |
| From *P*(*r*) | I(0) (cm^-1^) | 18.90 | 80.42 |
|  | Rg(Å) | 20.14 | 29.71 |
|  | Dmax (Å) | 66.0 | 98.3 |
| Molecular Mass Determination | | | |
| MM (kDa) from I(0) | | 22.1 | 73.8 |
| MM (kDa) from sequence | | 24.5 | 69 |
| Modeling | | | |
| DAMMIN | χ^2^ | 1.064 | 1.039 |
|  | NSD | 0.831±0.019 | 0.714±0.056 |
|  | Ensemble Resolution | 27±2 | 38±3 |
